# PD-L1 Amino Acid Position 88 Represents a Hotspot for PD-L1 Stability With Relevance for PD-L1 Inhibition

**DOI:** 10.3389/fonc.2022.941666

**Published:** 2022-07-22

**Authors:** Luise Victoria Claaß, Christoph Schultheiß, Rebekka Scholz, Lisa Paschold, Donjete Simnica, Volker Heinemann, Sebastian Stintzing, Mascha Binder

**Affiliations:** ^1^ Internal Medicine IV, University Hospital Halle, Martin-Luther University Halle-Wittenberg, Halle (Saale), Germany; ^2^ Department of Hematology/Oncology, Ludwig-Maximilians-University Munich (LMU) Klinikum, University of Munich and Comprehensive Cancer Center Munich, Munich, Germany; ^3^ Department of Hematology, Oncology, and Cancer Immunology (CCM), Charité - Universitätsmedizin Berlin, Berlin, Germany

**Keywords:** immune checkpoint blockade, PD-L1, avelumab, resistance, ddPCR

## Abstract

The two most common antibody targeting principles in oncology are the induction of direct antitumor effects and the release of antitumor T cell immunity by immune checkpoint blockade. These two principles, however, may be overlapping if the targeted checkpoint molecule is not located on the immune cell but on the tumor cell itself. Secondary resistance by epitope escape may therefore remain a challenge in both settings. We previously reported epitope escape through L88S and truncating programmed cell death ligand 1 (PD-L1) gene mutations in colorectal cancer patients on selective pressure with avelumab, a PD-L1-directed checkpoint blocker that—in addition to T cell disinhibition—allows direct tumor cell killing *via* its unmodified Fc portion. Here, we confirmed this principle by liquid biopsy monitoring in a colorectal cancer patient from an independent clinical trial. In this patient, both PD-L1 L88E and L88fs mutations emerged under selective pressure with avelumab. By ectopically expressing PD-L1 L88E, we show that this mutation leads to a reduction of full-length glycosylated PD-L1 and greatly reduced avelumab surface binding. Further experiments indicated that PD-L1 L88E represents a phosphomimetic variant of PD-L1 L88S leading to loss of protein stability and increased proteasomal degradation. The association of this PD-L1 mutation with the high-affinity *FCGR3A* single nucleotide polymorphism rs396991 confirms prior evidence that patients harboring this polymorphism experience the strongest selective pressure by avelumab. Together, position 88 of PD-L1 is a hotspot residue critically regulating PD-L1 cell surface expression with clinical significance in the context of immune checkpoint blockade.

## Introduction

Programmed cell death ligand 1 (PD-L1) is a membrane protein expressed on some cells of the tumor microenvironment such as macrophages and dendritic cells as well as on the surface of cancer cells (1, 2). It consists of IgV- and IgC-like domains, a trans-membrane, and a short intracellular domain. The interaction of the IgV domain of PD-L1 with its binding partner programmed cell death 1 (PD-1) on activated T cells triggers the formation of clusters with the T cell receptor and CD28, resulting eventually in abrogation of CD28 signaling by recruitment of Src homology 2 domain-containing tyrosine phosphatase 2 (SHP2) (3). As a result, T cells are inactivated and antitumor immune reactions dampened. To prevent tumor immune escape, a number of PD-1 and PD-L1 blocking antibodies have been developed and commercialized over the past decade resulting in significant therapeutic improvements for patients with a broad spectrum of tumors (4). Since PD-1 is expressed on the T cell itself, targeting antibodies like nivolumab or pembrolizumab were equipped with IgG4 constant regions showing reduced ability to bind complement or Fc gamma receptors on effector cells (5) to prevent the elimination of T cells by complement-dependent (CDC), antibody-dependent cellular cytotoxicity (ADCC), or antibody-dependent cell phagocytosis (ADCP) (6, 7). PD-L1 antibodies like durvalumab or atezolizumab harbor IgG1 instead of IgG4, but these regions have been genetically engineered to significantly reduce CDC or ADCC potentially directed not only at the tumor cell but also at PD-L1-positive cells within the tumor microenvironment (8, 9). In contrast, avelumab is a PD-L1 antibody with a functional IgG1 region that blocks the PD-L1/PD-1 axis, but may also directly promote ADCC against tumor cells (10). In a phase II trial where avelumab was given in addition to chemotherapy and EGFR-targeted therapy in a cohort of patients with predominantly microsatellite-stable metastatic colorectal cancer, we identified three patients with emerging tumor subclones on avelumab treatment that harbored *PD-L1* sequence variants (11). In two patients, we found a *PD-L1* L88S point mutation located outside the avelumab epitope resulting in lower membrane expression. A third patient developed a *PD-L1* K162fs variant inducing a premature stop codon, thereby resulting in complete loss of PD-L1 membrane expression due to a lack of the transmembrane domain. Cells transduced to express these variants triggered significantly less natural killer (NK) cell degranulation than cells expressing the PD-L1 wild-type, but T cell-mediated killing was increased. Interestingly, these *PD-L1* mutations were significantly associated with the homo- and heterozygous *Fc gamma receptor IIIa* (*FCGR3A*) single nucleotide polymorphism (SNP) rs396991 (V/V and V/F) that is known to mediate high-affinity binding of NK cells to IgG1 (12). This suggested that patients with microsatellite-stable metastatic colorectal cancer with the high-affinity *FCGR3A* SNP may be a subset with the highest selective pressure exerted by avelumab.

In the data presented here, we confirm the selection of two *PD-L1* mutants at position 88 on avelumab treatment in a patient with microsatellite-stable metastatic colorectal cancer. This patient—treated on an independent clinical trial—again showed homozygosity for the high-affinity *FCGR3A* SNP. We go on to show that *PD-L1* L88E represents a phosphomimetic mutation to *PD-L1* L88S leading to decreased protein stability and degradation. Together, our data suggest that position 88 may represent a clinically relevant *PD-L1* hotspot position regulating protein stability.

## Methods

### Patient and Biomaterial

Patient 05-001 was a patient enrolled on the FIRE-6 (AIO KRK-0118) trial with histologically confirmed, treatment-naive, RAS/RAF wild-type metastatic colorectal cancer (MSS) that agreed to take part in a biomarker substudy after informed consent [Ethical Commission of the Ludwig-Maximilians-University Munich (LMU): 18-0933]. The patient received 4 cycles of treatment with FOLFIRI and cetuximab, followed by 4 cycles of FOLFIRI/cetuximab with additional avelumab. These 8 cycles were followed by avelumab maintenance treatment for another 10 weeks until the end of treatment (EOT) due to progression in week 19. Plasma samples were collected at regular intervals in cell-free DNA BCT tubes (Streck, USA) and processed according to the manufacturer’s instructions. Plasma was immediately frozen at −80°C until extraction of cell-free DNA, and the leukocyte pellet was stored in 1 ml of heat-inactivated fetal bovine serum (Life Technologies, USA) with 10% of dimethyl sulfoxide (Sigma, Germany). Additionally, the paraffin-embedded tumor biopsy obtained before treatment initiation was used for genetic analyses as described below.

### Next-Generation Sequencing and Data Analysis

For mutational profiling, 100 ng of cell-free DNA (cfDNA) was used. Genes were selected through a review of published literature and by using the cBio Cancer Genomics Portal (13). Either the entire coding region or the hotspot regions containing known pathogenic or resistance variants were targeted, and sequencing libraries were constructed using QIAseq Targeted DNA Custom Panel (Qiagen, Germany). Quantification and quality control of the libraries were conducted using the Qubit high-sensitivity double-strand DNA assay kit (Thermo Fisher Scientific Inc., USA) and Agilent 2100 Bioanalyzer (Agilent Technologies, Germany). Sequencing was performed on the Illumina NextSeq or HiSeq platform (San Diego, USA) with 2 × 151 cycles at an average coverage of 13,500. Variant calling of unique molecular identifier (UMI)-based sequencing data was performed using the CLC workbench (Qiagen, Hilden, Germany). Variants were filtered for a minimum coverage of 10 UMIs, minimum count of 2 UMIs, minimal average base quality of 35, and minimum frequency of 1% for cfDNA or 10% for DNA from formalin-fixed paraffin-embedded tissue. Analyses were carried out and data plotting was performed using R (version 3.4.4) (14) as well as GraphPad Prism 7 (San Diego, CA). A *p*-value of <0.05 was considered statistically significant.

### Digital Droplet PCR Workflow

Digital droplet PCR (ddPCR) custom assays were designed by and ordered from IDT (Iowa, USA). To increase specificity, locked nucleic acid bases were incorporated into the probes. Probes were HPLC-purified and contained either a 5′-HEX (wild-type probe) or a 5′-FAM (mutant probe) reporter dye and a 3′Iowa Black^®^ Fluorescent quencher. The probe and primer sequences are listed in **Supplementary Table S1**. Each reaction (22 µl) contained 11 µl of 2×ddPCR SuperMix for probes (no UTP) (Bio-Rad, Feldkirchen, Germany), template DNA, and primers and probes at a final concentration of 1.8 µM and 500 nM, respectively. After droplet generation [AutoDG, Bio-Rad (Feldkirchen, Germany)], the plate was sealed with a pierceable foil heat seal [PX1 PCR plate sealer, Bio-Rad (Feldkirchen, Germany)], and the PCR was performed on a C1000 Touch thermal cycler (Bio-Rad, Feldkirchen, Germany). The cycling conditions were as follows: 95°C, 10 min; (94°C, 30 s; 56°C, 2 min] × 50; 98°C, 10 min; and 12°C hold for at least 4 h. Prior to the readout on the QX200 droplet reader (Bio-Rad, Feldkirchen, Germany), the plate was left at room temperature for 10 min. ddPCR data were analyzed using the QX Manager 1.0 Standard Edition (Bio-Rad, Feldkirchen, Germany). Assay specificity was tested on gBlocks (IDT, Iowa, USA). Thresholds for positive/negative droplets were set manually according to the positive control (gBlocks, IDT, **Supplementary Figure S1**).

### Generation and Evaluation of Cell Lines Stably Expressing PD-L1 Variants

For functional validation of *PD-L1* L88E, we used the UT-SCC-14 (tongue squamous cell carcinoma) and HT-29 (colorectal adenocarcinoma, *KRAS* wild-type) cell lines that were depleted of endogenous *PD-L1* by CRISPR/Cas9 as described in Stein et al. (11). The *PD-L1* L88E (c.262_263delinsGA) variant and L88M (c.262T>A) as control variants were generated by site-directed mutagenesis of the PD-L1 wt cDNA cloned into the Lentiviral Gene Ontology (LeGO) vector LeGO-iC2-Puro+ *via Asi*SI/*Eco*RI. Mutagenesis was performed using the QuikChange^®^ II XL Site-Directed Mutagenesis Kit according to the manufacturer’s instructions. For ectopic re-expression, the *PD-L1* constructs were lentivirally transduced and selected as described elsewhere (15). Cell lysates were generated using RIPA buffer supplemented with protease and phosphatase inhibitors (both from Roche, Basel, Switzerland) and quantified using the Pierce™ Detergent Compatible Bradford Assay Kit according to the manufacturer’s standard. For semidry immunoblotting, 20 µg of the extract was separated using the NuPAGE™ Bis-Tris gel system (Thermo Fisher, Hennigsdorf, Germany). The following antibodies were used for detection: anti-PD-L1, goat polyclonal IgG (R&D Systems, Minneapolis, United States); anti-GAPDH, mouse monoclonal IgG (Santa Cruz, Heidelberg, Germany); anti-goat-IgG-HRP, produced in mouse (Santa Cruz, Heidelberg, Germany); and anti-mouse-IgG-HRP, produced in goat (R&D Systems, Minneapolis, United States).

### qRT-PCR

Total RNA was extracted with the Quick-RNA kit (Zymo Research, Freiburg, Germany) and reverse-transcribed with SuperScript III (Thermo Fisher, Hennigsdorf, Germany). Target amplification was performed on the CFX96 System (Bio-Rad, Feldkirchen, Germany) using the SYBR Select Master Mix CFX as suggested by the manufacturer and using the following primer pairs: *PD-L1* (forward: TGCTGAACGCATTTACTGTCAC; reverse: TCTGTCTGTAGCTA CTATGCTG), *mCherry* (forward: AGGAGGATAACATGGCCATCAT; reverse: ACCC TTGGTCACCTTCAGCT), and *HPRT1* (forward: TGACACTGGCAAAACAATGCA; reverse: GGTCCTTTTCACCAGCAAGCT). The relative gene expression levels were normalized to *HPRT1* and calculated according to the comparative Ct (ΔΔCt) method.

### Flow Cytometry

To quantify PD-L1 surface expression, 100,000 cells per well were seeded on a 96-well plate and incubated with 100 ng of avelumab or a human IgG pool [Intratec^®^ (Biotest Pharma, Dreieich, Germany)] as isotype controls for 45 min at 4°C. After washing, avelumab was detected using a FITC-coupled α-human-IgG antibody (Sigma-Aldrich, Taufkirchen, Germany). Readout was performed on a BD FACSCelesta flow cytometer (Becton Dickinson, Kelberg, Germany) and analyzed using FlowJo™ (Becton Dickinson, Kelberg, Germany).

### PD-L1 Degradation Assays

To assess the turnover of PD-L1 variants, compound C/dorsomorphin and MG132 were obtained from Selleck Chem (Planegg, Germany). To analyze the degradation kinetics of PD-L1 variants, 2 million cells were seeded in a six-well format and either incubated with 10 µM of compound C for 2 and 4 h or with 20 µM of MG132 for 4 h. Proteins were isolated as described above and subjected to immunoblotting.

## Results

### Colorectal Cancer Patient With Acquired PD-L1 Mutations on Avelumab Treatment

**Figure 1:**
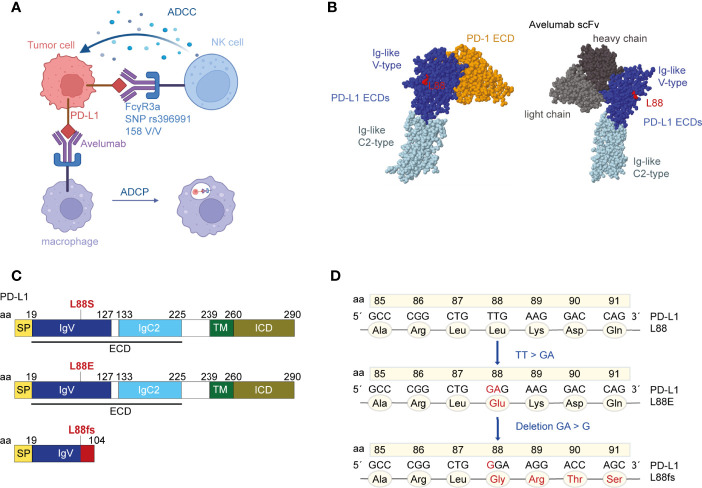
The localization of residue leucine 88 in the extracellular domains (ECDs) of programmed cell death ligand 1 (PD-L1). **(A)** Binding of avelumab to PD-L1 promotes antibody-dependent cell-mediated cytotoxicity **(ADCC)** or antibody-dependent cell phagocytosis (ADCP) against tumor cells *via* the Fcγ receptor. Patient 05-001 showed homozygosity for the *FCGR3A* SNP rs396991 (amino acid 158V/V, also known as 176V/V) that is known to mediate high-affinity binding of NK cells to IgG1 (12, 16). The figure was created with BioRender.com. **(B)** Crystal structure of PD-L1 ECDs in complex with PD-1 ECD [PDB ID: 3BIK (17)] and crystal structure of PD-L1 ECDs in complex with an avelumab single-chain variable fragment (scFv) [PDB ID: 5GRJ (18)]. Leucine 88 is highlighted in red. Space-filling models were created with RasMol (19). **(C)** Schematic structure of the human PD-L1 peptide with indications where the novel mutations theoretically introduce changes. PD-L1 L88fs leads to a premature termination codon and consecutively to loss of the transmembrane domain. **(D)** Nucleotide and amino acid exchanges found in patient 05-001 at week 9 and EOT in the PD-L1 L88 position.

The FIRE-6 trial combined chemotherapy with PD-L1 inhibition and EGFR inhibition in patients with metastatic colorectal cancer. The PD-L1 inhibitor avelumab was used which represents an antibody with preserved effector functions that have been previously shown to induce direct tumor cell killing, especially in subjects expressing the *FCGR3A* high-affinity SNP rs396991 (**Figure 1A**). We initially subjected 29 patients enrolled in the FIRE-6 trial to liquid biopsy testing at EOT using a gene panel covering the most frequent driver and resistance mutations in mCRC including the full coding region of *PD-L1*. We identified one patient with study ID 05-001 who showed a *PD-L1* L88E mutation at the end of treatment and profiled the patient consecutively with liquid biopsy at additional timepoints. While there was no PD-L1 L88 mutation at baseline, we found a *PD-L1* L88fs mutation at week 9 of the treatment protocol (**Figures 1B, C**, **Table 1**). Recapitulation of nucleotide exchanges suggested that the alteration had developed in several steps: at first, a TT to GA mutation (*PD-L1* L88E) followed by a GA to G deletion (*PD-L1* L88fs) leading to a premature stop codon at position 104 (**Figures 1C, D** **)**. Interestingly, patient 05-001 showed homozygosity for the *FCGR3A* high-affinity SNP rs396991 that was found associated with PD-L1 alterations in the AVETUX trial (11). To confirm the mutations detected by next-generation sequencing (NGS) with a more sensitive and specific method, we designed ddPCR primers for both genetic lesions and subjected all cfDNAs collected in patient 05-001 to serial ddPCR testing. This analysis confirmed the mutations identified *via* NGS showing comparable variant allele frequencies at week 9 and EOT (**Table 1**, **Supplementary Figure S1**), while baseline tumor tissue as well as germline leukocyte DNA was not mutated in this position. Notably, we did not detect acquired *EGFR* mutations mediating secondary resistance toward cetuximab.

**Table 1:** PD-L1 mutation frequencies in patient 05-001 across different timepoints.

Patient 05-001 samples	NGS frequency (%)		ddPCR fractional abundance (%)		Clinical response
	PD-L1 L88E	PD-L1 L88fs	PD-L1 L88E	PD-L1 L88fs	
Baseline leukocyte DNA			0	0	
Baseline tumor DNA			0	0	
cfDNA week 9	0	7.24	0	13.04	
cfDNA EOT	12.2	0	26.83	0	PD

### PD-L1 L88E Leads to Reduced Cellular PD-L1 Levels

**Figure 2:**
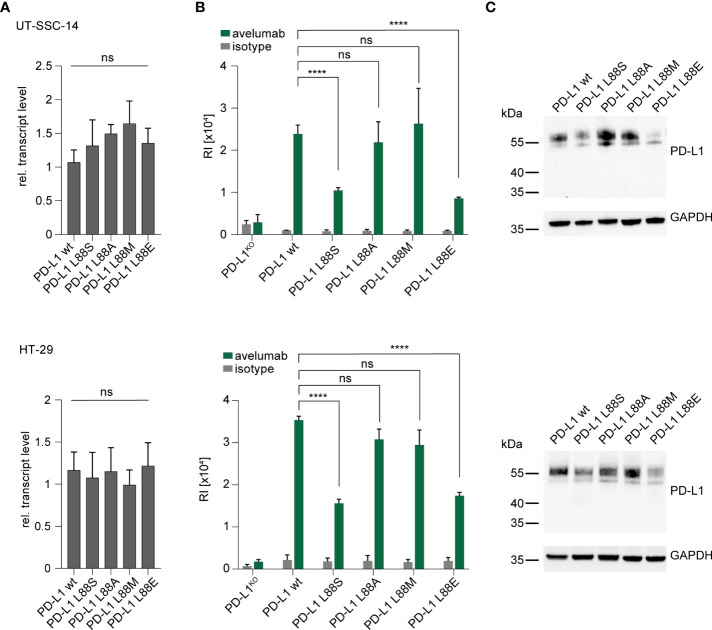
Introduction of new *PD-L1* variants into PD-L1-deficient tumor cells. (**A–C**) UT-SSC-14 and HT-29 cells ectopically expressing *PD-L1* L88S, L88A, L88M, and L88E were analyzed by **(A)** qRT-PCR (replicates *n* = 4), **(B)** flow cytometry (replicates *n* = 4-6), and **(C)** Western blotting in comparison with PD-L1 wt. Data are presented as mean ± SD. RI, relative mean fluorescence intensity. Statistics: Welch’s ANOVA over all groups **(A)** with *post-hoc* Dunnett’s test **(B)**. Asterisks indicate *p*-value range (*p < 0.05; **p < 0.01; ***p < 0.001; ****p < 0.0001; ns > 0.05).

As part of the extracellular domain, the *PD-L1* L88fs mutation leads to full abrogation of PD-L1 cell surface expression due to loss of the transmembrane domain (**Figure 1C**). This has been previously shown for a different frameshift mutation (*PD-L1* K162fs) that was equally localized in the extracellular domain (11). In contrast, the functional consequences of *PD-L1* L88E are unknown. We recently detected expanding subclones with *PD-L1* L88S mutations in two colorectal cancer patients from the AVETUX trial (11). The introduction of a new serine phosphorylation site diminished protein stability and fostered proteasomal degradation of PD-L1 (11). Since glutamic acid (E) or aspartic acid (D) can mimic the functional effects of a phosphorylated serine residue when replacing it in the phosphorylated target site (phosphomimetic mutations) (20), we hypothesized that this mechanism also applies to *PD-L1* L88E. To test this hypothesis, we lentivirally transduced a *PD-L1* L88E expression construct into the UT-SSC-14 and HT-29 cell lines that were depleted of endogenous PD-L1 expression by CRISPR/Cas9 (11). PD-L1 depletion of these cell lines did not affect their viability (11). As a control, the *PD-L1* wild-type as well as *PD-L1* L88A and L88M—two mutants without suspected phosphomimetic properties—was used. In addition, UT-SSC-14 and HT-29 cells overexpressing PD-L1 L88S served as control for enhanced degradation. While all cells expressing PD-L1 variants showed equal or slightly elevated transcript levels as compared to the wild-type (**Figure 2A**), protein abundancy varied depending on the expressed variant but with comparable patterns in flow cytometry and Western blotting: while the expression of the non-phosphomimetic L88A and L88M control mutants was equal (UT-SSC-14) or only minimally reduced (HT-29) as compared to the wild-type, both PD-L1 L88S and *PD-L1* L88E showed substantial reduction of surface PD-L1 (**Figure 2B**) as well as total PD-L1 levels in the cell (**Figure 2C**).

### PD-L1 L88E Induces Increased Proteasomal Degradation

**Figure 3:**
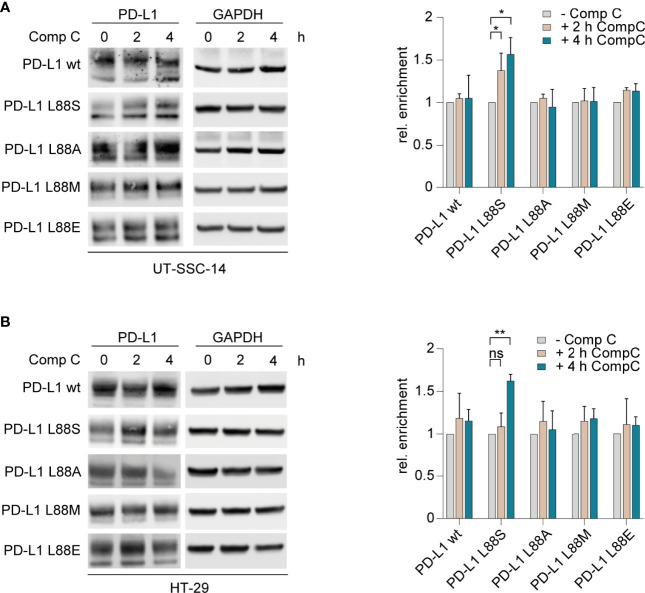
Inhibition of AMPK and examination of its effect on different PD-L1 variants. **(A)** UT-SCC-14 or **(B)** HT-29 cells overexpressing five different *PD-L1* variants were treated with compound C (CompC) to inhibit AMPK, and cell lysates were analyzed by Western blotting (*n* = 3–4) before and after 2 and 4 h of treatment. Band intensities of at least two independent experiments were quantified using ImageJ, and enrichment of PD-L1 was calculated relative to untreated controls after normalization to GAPDH (24). Data are presented as mean ± SD. Statistics: two-sided unpaired *t*-test. Asterisks indicate *p*-value range (*p < 0.05; **p < 0.01; ***p < 0.001; ****p < 0.0001; ns > 0.05).

The interplay of phosphorylation-induced degradation and glycosylation of PD-L1 is a key feature of homeostatic PD-L1 turnover and stability (21–23). To test the phosphomimetic potential of PD-L1 L88E, we first assessed whether L88E is reactive to inhibition of the AMP-activated protein kinase (AMPK) which was shown to phosphorylate serine on codon 88 of *PD-L1*, thus enhancing degradation (11). For this purpose, we treated the cell models with the AMPK inhibitor compound C (dorsomorphin) and quantified protein levels after 2 and 4 h using immunoblotting. As shown in **Figures 3A, B**, the protein levels of the PD-L1 wild-type as well as the L88A, L88M, and L88E variants were not affected by AMPK inhibition, while the amounts of PD-L1 L88S were enriched over time. This suggested that PD-L1 E88 was not phosphorylated by this kinase (**Figure 3**).

**Figure 4:**
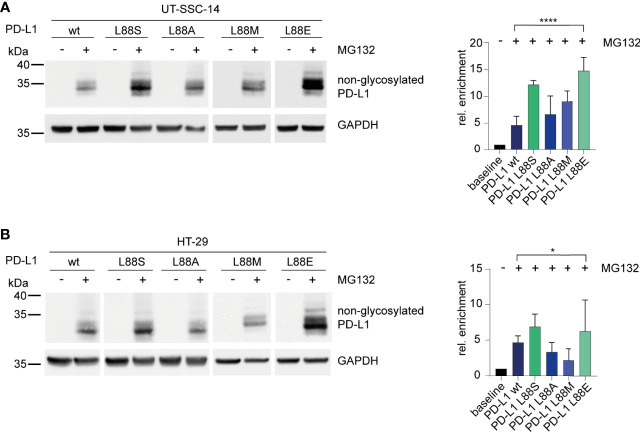
Inhibition of the 26S proteasome and examination of its effect on different PD-L1 variants. **(A)** UT-SCC-14 or **(B)** HT-29 cells overexpressing five different *PD-L1* variants were treated with MG132 to block the 26S proteasome, and cell lysates were analyzed by Western blotting before and after treatment. Band intensities of non-glycosylated PD-L1 derived from at least three independent experiments were quantified using ImageJ, and enrichment of PD-L1 was calculated relative to untreated samples (=baseline) after normalization to GAPDH concentrations (24). Data are presented as mean ± SD. Statistics: one-way ANOVA over all groups. Asterisks indicate *p*-value range (*p < 0.05; **p < 0.01; ***p < 0.001; ****p < 0.0001; ns > 0.05).

To confirm the phosphomimetic properties of the L88E mutation, we next investigated the degradation of the PD-L1 variants as readout. For this purpose, we treated the cell models with MG132, an inhibitor of the 26S proteasome, for 4 h. Immunoblotting revealed that PD-L1 L88S and L88E were enriched faster than the wild-type and the L88A and L88M variants (**Figures 4A, B** **)**.

## Discussion

We previously showed that direct tumor cell killing by ADCC can be considered a clinically relevant mechanism of action of avelumab, a PD-L1-directed immune checkpoint inhibiting antibody with preserved effector function (11). Secondary resistance by epitope escape—as seen in many cancer therapies targeted at a single antigen at the tumor cell surface [e.g (25–27).,]—is therefore not unexpected.

In the work presented here, we dissect the functional consequences of mutations at amino acid position 88 of PD-L1 that have been acquired in colorectal cancer patients across two clinical trials including the PD-L1 antibody avelumab as part of their experimental protocol. In the AVETUX and FIRE-6 studies, 72 patients were sequenced for the germline FCGR3A SNP as well as for PD-L1 mutations at baseline and end of treatment. None of the patients showed a PD-L1 mutation at baseline. In total, 3 of 7 patients that were homozygous for the FCGR3A SNP and 1 of 22 patients heterozygous for the FCGR3A SNP acquired a PD-L1 mutation on treatment. None of the 43 FCGR3A wild-type patients acquired a PD-L1 mutation in the course of avelumab treatment. The emergence of such mutations exclusively in patients expressing the *FCGR3A* V allele indicates that avelumab’s therapeutic pressure may largely be mediated by tumor-infiltrating innate immune effector cells, especially NK cells. NK cells from individuals with the SNP rs396991 (V/V and V/F) have been shown to have an especially high affinity to IgG (12, 28), thus enhancing their ability to mediate effective ADCC toward tumor cells coated with avelumab.

Two different alterations at position 88 of *PD-L1* have been identified in human patient samples by our group: an exchange from leucine to serine introducing a novel phosphorylation site that facilitates PD-L1 degradation and the exchange from leucine to glutamic acid. The experiments presented here demonstrate that E88 represents a phosphomimetic exchange with comparable consequences as S88 in terms of reduced PD-L1 protein stability and membrane expression resulting in significantly reduced avelumab binding. Notably, our data are in agreement with the post-translational regulation of PD-L1 stability which represents a key mechanism mediating its biological functions (21, 22, 29, 30). The stability of PD-L1 is conferred by its glycosylation (asparagine residues 192, 200, and 219) which masks targeted phosphosites (threonine 180, serine 184, serine 195) and/or prevents the binding of several kinases like GSK3β (21), CDK4/6 (22), or AMPK (29) which phosphorylate PD-L1 and thus induce its degradation *via* the proteasome or the ER-associated protein degradation (ERAD). The acquisition of another phosphorylated serine outside this region allows glycosylation-independent phosphorylation-induced proteasomal degradation. In contrast, the *PD-L1* L88fs leads to full PD-L1 loss.

The detection of *PD-L1* L88fs at an earlier timepoint (week 9) than *PD-L1* L88E (detected at EOT) appeared counterintuitive since our nucleotide sequence analysis suggested that the L88fs variant emerged from the L88E variant. Interestingly, the “precursor” variant L88E was undetectable both by NGS and ddPCR at week 9. It appears likely that this is rather a matter of sensitivity of detection, but it shows that this variant—at this point in treatment—may have shown a lower biological fitness than the full PD-L1 loss variant L88fs. The strong selective advantage of the L88fs variant in the early treatment phase was likely due to its potent escape from direct antitumor pressure exerted by avelumab. In later phases, this selective advantage might have been counterbalanced by a higher T cell pressure suppressing this PD-L1-negative clone. In the long run, the reduced but not completely absent PD-L1 expression of clone *PD-L1* L88E may have offered protection from avelumab’s direct antitumor effects while at the same time counteracting T cell pressure. Notably, other therapeutic anti-PD-L1 antibodies like atezolizumab and durvalumab have IgG regions specifically designed to avoid effector functions (8, 9). Since they only function as blockers of the PD-1/PD-L1 interaction, it is very likely that patients treated with these antibodies will not select PD-L1 escape mutations as observed under avelumab treatment.

From a tumor biological perspective, these data are interesting since they show the complexity of subclonal selection principles with drugs that have different mechanisms of action. Under such conditions, only tumor subclones with a multidimensional escape to different selective forces will establish themselves successfully in the long run. On the other hand, our data show the importance to target surface molecules and epitopes that are of biological importance for the tumor. If this is not the case, the tumor will abrogate surface expression or express an epitope-disrupted but still functional receptor as has been previously shown for EGFR antibodies and CD19-targeting cell therapies (25–27, 31, 32). The finding of a close association between *FCGR3A* V allele expression and PD-L1 escape mutation suggests that the selective direct antitumor pressure needs to be extremely high to allow for the selection of such mutants that—on the other hand—show compromised biological fitness due to reduced inactivation of T cells.

Taken together, our mutational data from cancer patients and the subsequent dissection of functional consequences of these mutations show that amino acid position 88 of PD-L1 represents a hotspot that critically influences PD-L1 membrane expression. These data have relevance for immune checkpoint blockade in solid tumors using PD-L1 antibodies with preserved effector functions.

## Data Availability Statement

The datasets presented in this study can be found in online repositories. The names of the repository/repositories and accession number(s) can be found below: https://www.ebi.ac.uk/ena, PRJEB51424.

## Ethics Statement

The studies involving human participants were reviewed and approved by Ethical Commission of the Ludwig-Maximilians-University Munich (LMU): 18-0933. The patients/participants provided their written informed consent to participate in this study.

## Author Contributions

Conception and design: LC, CS, and MB. Collection and assembly of data: VH, SS, LC, CS, RS, LP, and DS. Data analysis and interpretation: LC, CS, RS, LP, and DS. Manuscript writing and final approval of the manuscript: all authors.

## Funding

This work was funded by Deutsche Krebshilfe (70114663 to MB).

## Conflict of Interest

SS received honoraria for talks and advisory board role from Amgen, Astra-Zeneca, Bayer, BMS, ESAI, LEO Pharma, Lilly, Merck KGaA, MSD, Pierre-Fabre, Roche, Sanofi, Servier, Taiho, and Takeda and research funding from Merck KGaA, Pierre-Fabre, Servier, and Roche. VH received honoraria for talks and advisory board role from Merck, Amgen, Roche, Sanofi, Sirtex, Servier, Pfizer, Pierre-Fabre, AstraZeneca, BMS, MSD, Novartis, Boehringer Ingelheim, Pierre-Fabre, Celgene, Terumo, Oncosil, and Seagen and research funding from Merck, Amgen, Roche, Sanofi, Pfizer, Boehringer-Ingelheim, Sirtex, and Servier.

The remaining authors declare that the research was conducted in the absence of any commercial or financial relationships that could be construed as a potential conflict of interest.

## Publisher’s Note

All claims expressed in this article are solely those of the authors and do not necessarily represent those of their affiliated organizations, or those of the publisher, the editors and the reviewers. Any product that may be evaluated in this article, or claim that may be made by its manufacturer, is not guaranteed or endorsed by the publisher.
