# Supplementary material for: PD-L1 Amino Acid Position 88 Represents a Hotspot for PD-L1 Stability With Relevance for PD-L1 Inhibition
Source: Front Oncol. 2022 Jul 22;12:941666. doi: 10.3389/fonc.2022.941666 (PMC9353709; doi:10.3389/fonc.2022.941666)
Supplement: Supplementary file 1 [file DataSheet_1.docx]

Supplementary Material

# Supplementary Table

**Supplementary Table S1**. ddPCR primer and probe sequences.

| **Assay** |  |  | **Sequence 5´to 3´** | **5´Reporter dye** |
| --- | --- | --- | --- | --- |
| **PD-L1**  **L88E** | forward | primer | CATGGAGAGGAAGACCTGAA |  |
|  | reverse | primer | CACATCTGTGATCTGAAGTGC |  |
|  | wild-type | probe | CTG+T+T+GAA+G+G+AC | HEX |
|  | mutant | probe | CTG+G+A+GAA+G+GAC | FAM |
| **PD-L1**  **L88fs** | forward | primer | CATGGAGAGGAAGACCTGAA |  |
|  | reverse | primer | CACATCTGTGATCTGAAGTGC |  |
|  | wild-type | probe | CTG+T+T+GAA+G+G+AC | HEX |
|  | mutant | probe | CT+G+G+GAA+GG+AC | FAM |

Base after “+” is a locked nucleic acid base

## Supplementary Figure


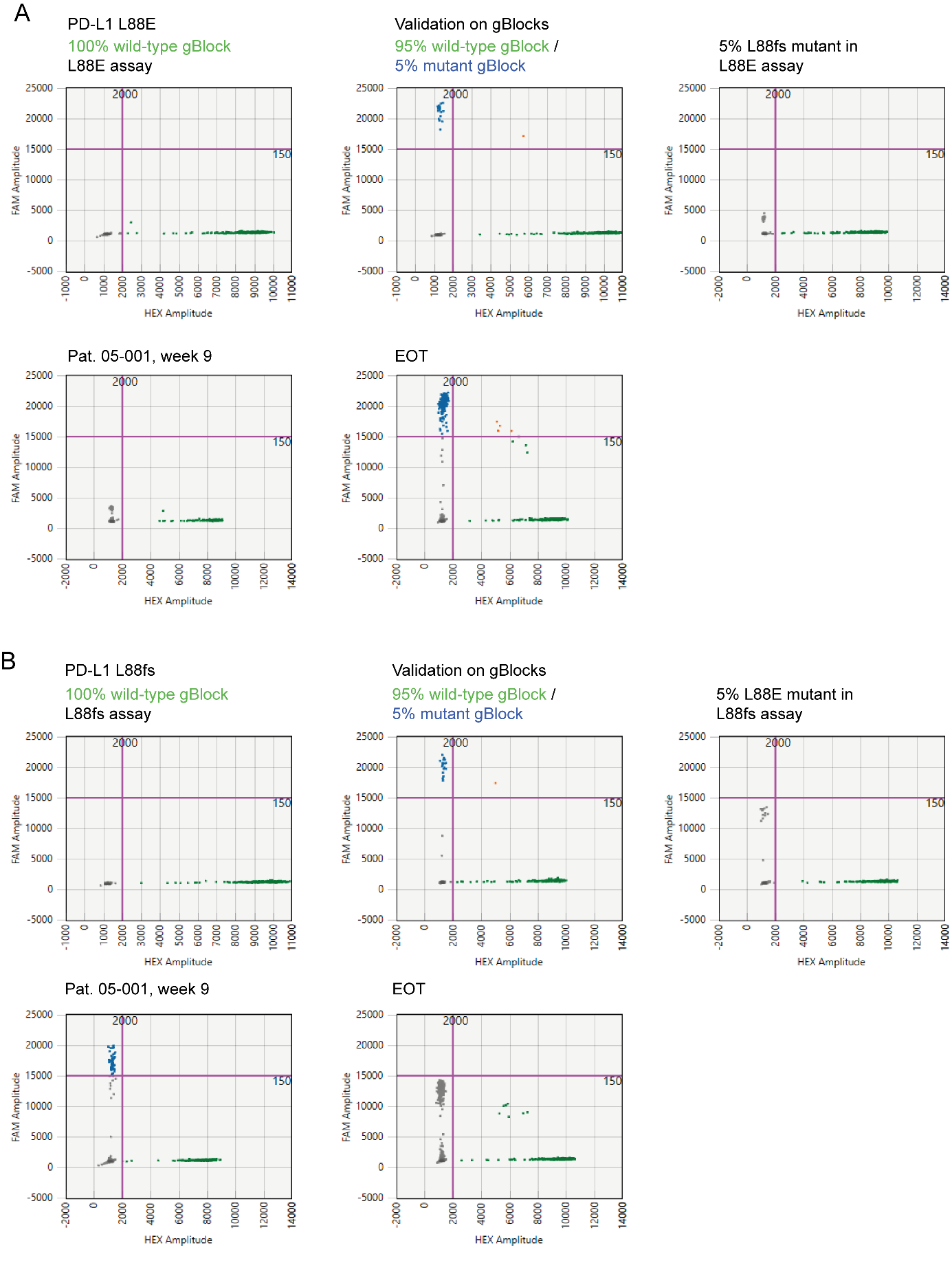


**Supplementary** **Figure S1.** Two-Dimensional plots for each ddPCR assay. **A,** PD-L1 L88E, **B,** PD-L1 L88fs. X-axis: HEX amplitude, Y-axis: FAM amplitude. Pink lines with adjacent number indicate thresholds for discrimation of positive/negative droplets. Top line: Each assay was validated on gBlocks with the wild type or mutant sequence. Additionally, the incongruent gBlock was used to demonstrate the possible discrimation between mutations by the assay. Bottom line: representative 2D plot for patient with respective assay at week 9 and EOT.
